# Morphology and mitochondrial genome-based analysis of the systematics and evolution of *Acanthochitona* species (Polyplacophora: Acanthochitonidae)

**DOI:** 10.1007/s42995-026-00362-9

**Published:** 2026-02-16

**Authors:** I Hyang Kim, Ui Wook Hwang

**Affiliations:** 1 Department of Biomedical Convergence Science and Technology, School of Industrial Technology Advances, Kyungpook National University, Daegu, 41566 Korea; 2 Department of Advanced Bioconvergence, Kyungpook National University, Daegu, 41566 Korea; 3 Department of Biology Education, Teachers College & Institute for Phylogenomics and Evolution, Kyungpook National University, Daegu, 41566 Korea; 4 Institute for Korean Herb-Bio Convergence Promotion, Kyungpook National University, Daegu, 41566 Korea; 5 Phylomics Inc., Daegu, 41544 Korea

**Keywords:** Chiton, *Acanthochitona*, Mitochondrial genome, Morphology, Molecular phylogeny, Divergence time

## Abstract

Chitons, known as marine living fossils, have retained their ancestral traits for approximately 300 million years. The genus *Acanthochitona* (Polyplacophora: Acanthochitonidae), characterized by the presence of 9 pairs of sutural tufts on a well-expanded girdle, is distributed across the intertidal zones of South Korea, Japan, China, and the Indo-Pacific. This study examined five *Acanthochitona* species from South Korea: *A*. *achates*, *A*. *circellata*, *A*. *defilippii*, *A*. *rubrolineata*, and *A*. *feroxa* sp. nov. Their mitochondrial genome sequences ranged from 14,986 to 15,006 bp in length and with a gene content typical for Polyplacophora. Genetic (including a transitive consistency score [TCS] genetic network), principal coordinate, phylogenetic network, and *CO1-*based barcoding gap analyses confirmed a new species, *A*. *feroxa* sp. nov., which exhibited morphologically distinct dorsal spicules and radulae. Maximum likelihood (ML) and Bayesian inference (BI) trees were constructed based on the *CO1* sequences of 28 polyplacophoran species belonging to 9 families, which placed these five *Acanthochitona* species within a monophyletic family, Acanthochitonidae. The analyses also indicated the polyphyletic nature of Mopaliidae, recommending a reclassification. Divergence time estimation revealed that *Acanthochitona* deviated during the Late Cretaceous (ca. 83.94 mya), with continued speciation occurring in the Paleogene and Neogene periods. Additionally, we constructed a pictorial key based on the ML tree for morphologically identifying the five *Acanthochitona* species. This study contributes to the understanding of speciation and phylogenetic relationships within the Acanthochitonidae, offering valuable insights into the classification scheme and mitochondrial genome evolution of chitons in the western Pacific.

## Introduction

Members of the class Polyplacophora, commonly known as chitons, represent one of the most ancient and primitive groups within the phylum Mollusca. Chitons are characterized by bilateral symmetry and a dorsoventrally flattened body, typically oval, elongated–oval, or vermiform in shape. The dorsal surface is covered by eight overlapping valves, encircled by a girdle that bears scales, spicules, spines, hairs, needles, or bristles (Okusu et al. 2003). To date, ~ 940 extant (Schwabe 2005; Stebbins and Eernisse 2009) and 430 fossil (Puchalski et al. 2008) species of chitons have been recorded. They are distributed globally, from tropical to polar waters, and inhabit environments ranging from intertidal zones to the deep-sea regions. They typically attach to substrates such as rocks, shells, or other organisms and primarily feed on algae (Jörger et al. 2008; Sigwart and Schwabe 2017). The ancestral history and ecological characteristics of chitons have remained largely unchanged for approximately 300 million years, with no significant morphological differences between the fossil and extant species. Thus, they are considered living fossils. Such an evolutionary stability positions chitons as one of the molluscan groups with the most primitive evolutionary history, granting them a unique place in the study of molluscs (Scherholz et al. 2013).

Sirenko (2006) classified modern chitons into two orders: Lepidopleurida and Chitonida, with the latter being further divided into the suborders Chitonina and Acanthochitonina based on their morphological features. The genus *Acanthochitona* (family Acanthochitonidae, suborder Acanthochitonina) contains 84 species, six of which have been found in South Korea (Hong et al. 1990). *Acanthochitona* J. E. Gray, 1821, is morphologically distinct from other chiton groups. It is characterized by a reduced tegmental area and merged pleural and lateral regions of the shell plates, the dorsal side of which is typically covered by raised granules. The plates exhibit five slits on the ventral side of the head, with a single slit on each side of the intermediate plates and usually two slits in the tail valve (Watters 1990). Their most conspicuous feature is the presence of 18 tufts of aragonitic bristles on the perinotum in most species.

The classification of chitons has traditionally relied on morphological features, such as valve structure, articulamentum, pustules, and the radula (Schwabe 2005). While this approach is widely used, concerns about potential taxonomic errors have arisen due to individual variations in these traits, which can lead to misidentification. *Acanthochitona* exhibits remarkable variability in these morphological features, challenging species identification due to substantial similarities and differences (Bonfitto et al. 2011). In recent years, the mitochondrial genome has emerged as a robust target for the molecular identification of species, enhancing taxonomic accuracy. These genomes exhibit a maternal inheritance pattern and short mutation fixation times within populations, making them highly useful for detecting genetic variations (Irisarri et al. 2020). They have been used across various scientific disciplines, including molecular phylogenetics and population genetics (Akintola et al. 2022; Choi and Hwang 2023; Choi et al. 2021a; Kim et al. 2019, 2024; Lee et al. 2012; Park et al. 2021). The mitochondrial genome has a circular double-stranded structure and consists of 37 genes, including 13 protein-coding genes (PCGs), two ribosomal RNA (rRNA) genes, and 22 transfer RNA (tRNA) genes. Notably, the mitochondrial gene, cytochrome *c* oxidase subunit 1 (*CO1*), has served as an effective molecular marker for species identification employing DNA barcoding systems (Choi et al. 2021b; Hong et al. 2023; Kim and Hwang 2023; Shin et al. 2021).

This study investigated four previously recognized *Acanthochitona* species: *A*. *achates*, *A*. *circellata*, *A*. *defilippii*, and *A*. *rubrolineata*; it also described a new species, *A*. *feroxa* sp. nov. To address the taxonomic challenges, we employed an integrated approach combining molecular and morphological analyses. The genetic distinctiveness of *A*. *feroxa* sp. nov. was evaluated through *CO1* barcoding and morphological differences. The mitochondrial genomes of all five species were completely sequenced, and features such as gene arrangement, nucleotide composition, and codon usage pattern were compared. Phylogenetic relationships within Polyplacophora were inferred using the maximum likelihood (ML) and Bayesian inference (BI) methods, and based on the 13 mitochondrial PCG sequences. Additionally, a pictorial key was constructed based on morphological characteristics and the ML tree to facilitate species identification in a user-friendly manner. Finally, the divergence times for *Acanthochitona* within Polyplacophora were estimated using a molecular clock model. The findings of this study can provide foundational data for future molecular investigations into *Acanthochitona*, offering insights into the complete mitochondrial genomes of these five species and their phylogenetic relationships.

## Materials and methods

### Sample collection

*Acanthochitona* specimens were collected from the intertidal zone of the South Korean coast. All samples were preserved in 99% ethanol and deposited in the sample collection of the Institute for Phylogenomics and Evolution, Kyungpook National University, Daegu, South Korea (Prof. Ui Wook Hwang, uwhwang@knu.ac.kr). Collection location details and the voucher numbers are provided in Tables 1 and 2. The samples were initially identified to the species level based on their external morphological characteristics and compared with the other *Acanthochitona* species’ data from a previous study (Hong et al. 1990).

**Table 1:** Collection locations, with GPS coordinates, of the five *Acanthochitona* species used for mitochondrial genome sequencing in this study, along with their voucher numbers and the GenBank accession numbers of their mitogenomes

Species	Locality (South Korea)	GPS		Voucher number	GenBank accession number
		Latitude	Longitude		
*Acanthochitona achates*	Pohang, Gyeongsangbuk-do	35°58 ‘N	129°32 ‘E	LEGOM040554	PQ301026
*Acanthochitona circellata*	Shinan, Jeollanam-do	34°50 ‘N	126°17 ‘E	LEGOM040552	PQ301027
*Acanthochitona defilippii*	Seogwipo, Jeju-do	33°14 ‘N	126°32 ‘E	LEGOM040550	PP419021
*Acanthochitona rubrolineata*	Hampyeong, Jeollanam-do	35°5 ‘N	126°26 ‘E	LEGOM040553	PQ301028
*Acanthochitona feroxa* sp.nov	Tongyeong, Gyeongsangnam-do	34°52 ‘N	128°24 ‘E	LEGOM040549	OR757114

**Table 2:** Collection locations, with abbreviations and GPS coordinates; numbers of individuals; GenBank accession numbers of *CO1* sequences; and references for the *A*. *defilippii*, *A*. *rubrolineata*, and *A*. *feroxa* sp. nov. samples used for the *CO1*-based genetic analyses, including the TCS network, principal component, phylogenetic network, and DNA barcoding analyses

Species	Collection locality		Abbreviations	GPS		Number of individuals	GenBank accession number(s)	Reference(s)
				Latitude	Longitude		*CO1*	
*Acanthochitona defilippii*	South Korea	Seogwipo, Jeju-do	JJ	33°14’N	126°32’E	11	PQ651609–PQ651619	This study
		Taean, Chungcheongnam-do	TA	36°49’N	126°9’E	2	HM180425, HM180426	Kim et al. 2012
		Unknown	UN	**–**	**–**	2	OL877115, OL877116	Unpublished
	Japan	Miura, Kanagawa, Honshu	KG	35°12’N	139°22’E	3	MG680031, MG680032, MG680034	Eernisse et al. 2018
		Manazuru, Kanagawa, Honshu		35°9’N	139°8’E	2	MG680033, LC214417	Eernisse et al. 2018, Owada 2023
*Acanthochitona rubrolineata*	South Korea	Masan, Gyeongsangnam-do	MA	35°6’N	128°31’E	10	PQ775079–PQ775088	This study
		Goheung, Jeollanam-do	GH	34°32’N	127°14’E	21	PQ775058–PQ775078	This study
		Yeosu, Jeollanam-do	YS	34°40’N	127°45’E	3	PQ775055, PQ775056, PQ775057	This study
				34°47’N	127°45’E	20	MN205833–MN205852	Ni et al. 2020
		Taean, Chungcheongnam-do	TA	36°23’N	126°250’E	20	MN205791–MN205810	Ni et al. 2020
		Ansan, Gyeonggi-do	AS	37°11’N	126°32’E	20	MN205753–MN205770	Ni et al. 2020
		Buan, Jeollabuk-do	BA	35°37’N	126°27’E	20	MN205771–MN205790	Ni et al. 2020
		Wando, Jeollanam-do	WD	34°19’N	126°44’E	22	MN205811–MN205832	Ni et al. 2020
	Japan	Kinaisemen, Kyushu	KI	33°22’N	129°51’E	7	MN205853–MN205859	Ni et al. 2020
		Nabegushimen, Kyushu	NB	33°24’N	129°47’E	8	MN205860–MN205867	Ni et al. 2020
		Nomomachi, Kyushu	NM	32°35’N,	129°45’E	3	MN205868, MN205869, MN205870	Ni et al. 2020
	China	Weihai, Shandong	WH	37°32’N	122°09’E	20	MN205871–MN205890	Ni et al. 2020
		Lianyungang, Jiangsu	LY	34°45’N	119°29’E	20	MN205891–MN205910	Ni et al. 2020
		Qingdao, Shandong	QD	36°02’N	120°21’E	20	MN205911–MN205930	Ni et al. 2020
*Acanthochitona feroxa* sp. nov	South Korea	Tongyeong, Gyeongsangnam-do	TY	34°52’N	128°22’E	33	PQ651620–PQ651652	This study
		Yeosu, Jeollanam-do	YS	34°52’N	128°24’E	25	PQ651653–PQ651677	This study
		Seogwipo, Jeju-do	JJ	33°14’N	126°32’E	2	PQ651678, PQ651679	This study
		Taean, Chungcheongnam-do	TA	36°49’N	126°9’E	1	HM180423	Kim et al. 2012

### DNA extraction and PCR amplification

Genomic DNA was extracted from the foot of each specimen using a DNeasy Blood & Tissue Kit (Qiagen, Hilden, Germany). Its quality and quantity were checked with a Nanodrop4000 spectrophotometer (Thermo Fisher Scientific, MA, USA). The partial *CO1* sequence was PCR-amplified utilizing the universal primer set: LCO1490 (5′-GGT CAA CAA ATC ATA AAG ATA TTG G-3′) and HCO2198 (5′-TAA ACT TCA GGG TGA CCA AAA AAT CA-3′) (Folmer et al. 1994). The PCR mix consisted of 40.75 μL of distilled water, 5 μL of 10 × Taq DNA polymerase buffer, 1 μL of dNTP mixture (2.5 mmol/L each), 1 μL of each primer, 0.25 μL of Taq DNA polymerase, and 1 μL of genomic DNA. The thermocycler program included an initial denaturation step at 94 °C for 1 min; 35 consecutive cycles of denaturation at 94 °C for 1 min, annealing at 48 °C for 1 min, and elongation at 72 °C for 1 min; and a final elongation step at 72 °C for 10 min. The amplicon size was confirmed through 1% agarose gel electrophoresis. The PCR products were sequenced employing a BigDye Termination Sequencing Kit (PerkinElmer Co., CT, USA) on an ABI Prism 3730 DNA sequencer (PerkinElmer). The *CO1* sequences were uploaded to the GenBank database of the national center for biotechnology information (NCBI) and are available under the accession numbers PQ651609–PQ651679.

### Mitochondrial genome sequencing

Mitochondrial genomes of the four *Acanthochitona* species were sequenced using next-generation sequencing (NGS). The *A*. *achates*, *A*. *defilippii*, and *A*. *rubrolineata* genomes were sequenced utilizing an Illumina TruSeq library with an average insert size of 350 bp and the TruSeq DNA Nano 350 bp kit (Illumina, CA, USA). For *A*. *circellata*, the TruSeq DNA Nano 550 bp kit (Illumina) was employed. After preparation, the library was sequenced on the Illumina NovaSeq 6000 platform at DNA Link Inc., Seoul, South Korea, producing 151 bp long paired-end reads. The sequences were assembled employing NOVOPlasty 4.3.5. (Dierckxsens et al. 2017) with the mitochondrial genome of *A*. *avicula* (Irisarri et al. 2020) used as a reference.

The complete mitochondrial genome of *A*. *feroxa* sp. nov. was PCR-amplified using two universal primer pair sets: LCO1490 (5′-GGT CAA CAA ATC ATA AAG ATA TTG G-3′; Folmer et al. 1994) and 16Sar (5′-CGC CTG TTT ATC AAA AAC AT-3′; Palumbi et al. 1991) as well as HCO2198 (5′-TAA ACT TCA GGG TGA CCA AAA AAT CA-3′; Folmer et al. 1994) and 16Sbr (5′-CCG GTC TGA ACT CAG ATC ACG T-3′; Palumbi et al. 1991) to amplify the *CO1* of the *16S rRNA* fragment. The amplicon size was confirmed through 1% agarose gel electrophoresis, and Sanger sequenced using a BigDye Termination Sequencing Kit (PerkinElmer) on an ABI Prism 3730 DNA sequencer (PerkinElmer).

### Gene annotation

**Fig. 1:**
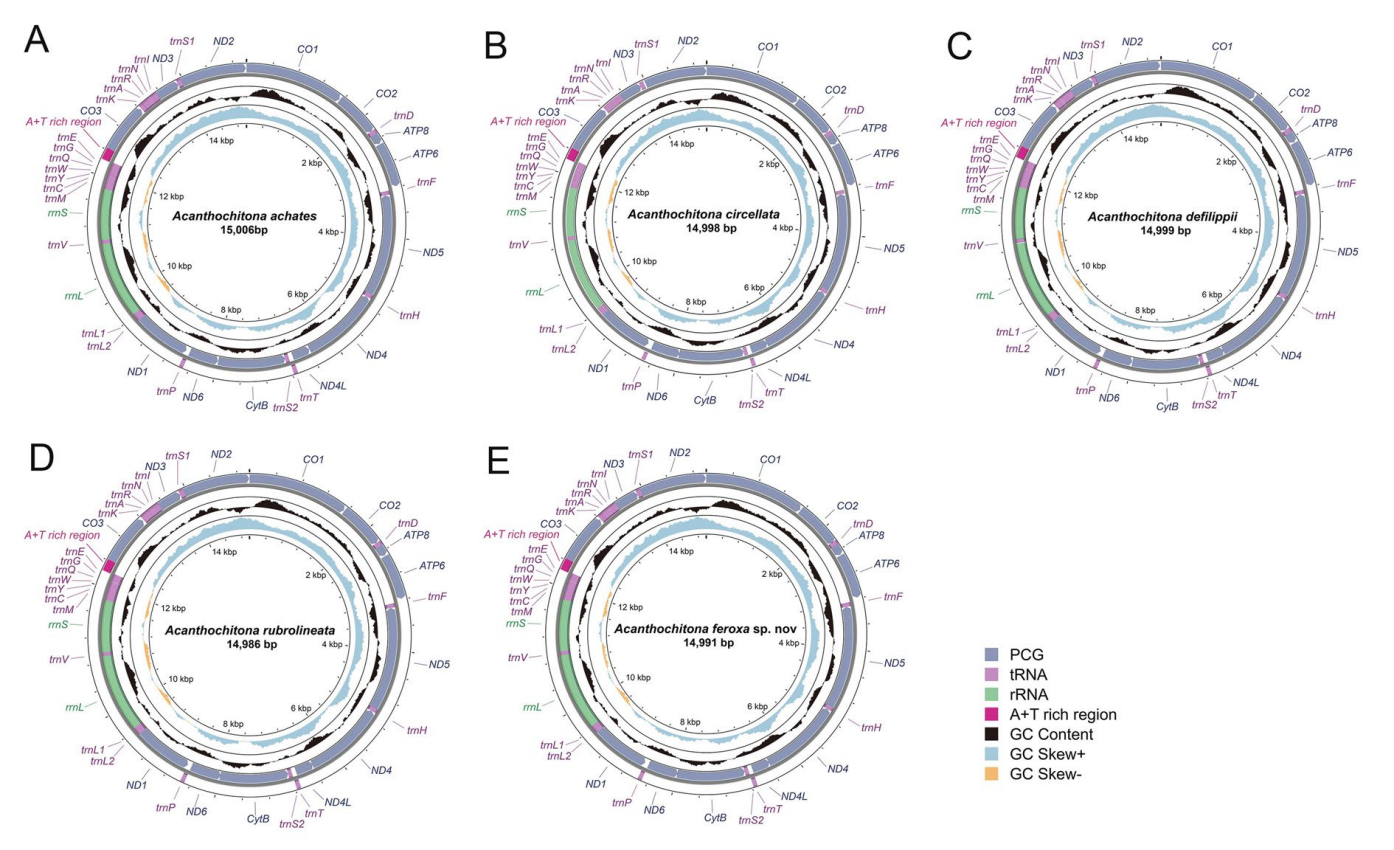
Circular maps of the mitochondrial genomes from five *Acanthochitona* species: **A** *A*. *achates*, **B** *A*. *circellata*, **C** *A*. *defilippii*, **D** *A*. *rubrolineata*, and **E** *A*. *feroxa* sp. nov. The PCGs located on the heavy strand include *ATP6*, *ATP8*, *COX1–3*, *ND2*, and *ND3*, while those on the light strand are *CytB*, *ND1*, *ND4*, *ND4L*, and *ND6*. The middle black circle displays the GC content, and the paracentral orange–light blue circle indicates the GC skew. The maps were visualized using the Proksee program (Grant et al. 2023)

A total of 13 PCGs and two rRNA genes were predicted employing the EMBOSS Transeq tool (Madeira et al. 2022) and accessing the MITOS2 web server (http://mitos2.bioinf.uni-leipzig.de/index.py; Donath et al. 2019). Additionally, 22 tRNA genes were predicted utilizing the tRNAscan-SE program (Chan and Lowe 2019) and the ARWEN software (Laslett and Canbäck 2008). After annotation, the circular mitochondrial genomes of the five *Acanthochitona* species were visualized (Fig. 1) using the Proksee system (Grant et al. 2023). The mitochondrial genome sequences were uploaded to the GenBank database and are listed under the accession numbers PQ301026, PQ301027, PP419021, PQ301028, and OR757114 for *A*. *achates*, *A*. *circellata*, *A*. *defilippii*, *A*. *rubrolineata,* and *A*. *feroxa* sp. nov., respectively.

### Population genetic analyses

To examine the genetic divergence among *A*. *defilippii*,* A*. *rubrolineata*, and* A*. *feroxa* sp. nov., we used the mitochondrial *CO1* sequences from 295 individuals. Of these, 105 were collected during this study, while the remaining 190 were retrieved from the NCBI database (Table 2). The sequences were aligned using Biodeit (Hall 1999) based on the ClustalW alignment method (Thompson et al. 1994). The number of polymorphic sites and haplotypes, as well as haplotype and nucleotide diversity (Table 3), for each population were estimated employing the DNA sequence polymorphism (DnaSP) v6.0 software package (Rozas et al. 2017). A haplotype network was constructed employing a statistical parsimony approach at the population level with the PopART software (Clement et al. 2002). To further evaluate and visualize the geographic variations and genetic structure among the populations, an unrooted phylogenetic network based on the neighbor-net algorithm was reconstructed using the splitstree software (Huson and Bryant 2006). A principal coordinate analysis (PCoA) was performed in the darwin 6.0.9 program (Perrier and Jacquemoud-Collet 2006) to visualize the sequence variations.

**Table 3:** Population genetics and neutrality test statistics of the 295 *CO1* sequences from *A*. *defilippii, A*. *rubrolineata*, and *A*. *feroxa* sp. nov. samples belonging to 17 populations

Population	*N*	*N*_h_	*h*	*π*	*S*	*k*	Tajima’s *D*	Fu’s *Fs*
TY	33	5	0.456	0.00114	4	0.58333	− 1.02197	− 1.968
YS	48	29	0.944	0.04363	75	22.25000	1.00017	− 1.320
JJ	13	7	0.846	0.02768	47	14.11538	− 0.39112	3.496
TA	23	12	0.874	0.02213	62	11.28854	− 1.37818	1.023
UN	2	1	0.000	0.00000	0	0.00000	0.00000	0.000
KG	5	4	0.900	0.00706	8	3.60000	− 0.44037	− 0.036
MA	10	6	0.778	0.00423	10	2.15556	− 1.72953	− 1.637
GH	21	10	0.686	0.00465	11	2.37143	− 0.78570	− 3.579
AN	20	6	0.632	0.00524	13	2.67368	− 0.98064	0.559
BA	20	13	0.932	0.00765	17	3.90000	− 0.69615	− 5.115
WD	22	12	0.861	0.00693	18	3.53680	− 1.04372	− 3.771
KI	7	7	1.000	0.01083	15	5.52381	− 0.54049	− 2.773
NB	8	6	0.929	0.01015	14	5.17857	− 0.20829	− 0.411
NM	3	3	1.000	0.00654	5	3.33333	− 0.077	0.000
WH	20	8	0.837	0.00850	16	4.33684	− 0.14263	0.301
LY	20	7	0.711	0.00563	16	2.87368	− 1.34846	− 0.119
QD	20	5	0.600	0.00389	13	1.98421	− 1.66317	0.667

Diversity statistics are provided for each locality: *N* the number of *CO1* sequences (individuals), *N*_h_ the number of haplotypes, *h* haplotype diversity, *π* Jukes–Cantor-corrected estimates of the nucleotide diversity, *S* the number of segregation sites, and k the average number of pairwise nucleotide differences. Statistical significance values: *P < 0.05 and **P < 0.01. No statistically significant variations were observed within the current dataset. The abbreviations of the locations from where the population samples were collected are defined in Table 2

### Barcoding and molecular species delimitation

The *CO1-*based barcoding gaps were analyzed employing the online version of the automatic barcode gap discovery (ABGD) method (https://bioinfo.mnhn.fr/abi/public/abgd/abgdweb.html) to generate distance histograms, distance ranks, and automatic partitions. These analyses were conducted using the Kimura 2-P distance matrix and 2 parameters: the prior intraspecific divergence ranging from *P*_min_ (0.001) to *P*_max_ (0.1) and the relative gap width (*X* = 1.5). Species delimitation analyses were performed using the Assemble Species by Automatic Partitioning (ASAP) program (Puillandre et al. 2021) and Bayesian Poisson Tree (bPTP) processes (Zhang et al. 2013). ASAP was conducted employing the Kimura K80 substitution model (*t*s/*t*v = 2.0) via the web server available at https://bioinfo.mnhn.fr/abi/public/asap. Species delimitation was conducted via the bPTP web server (https://species.h-its.org/). Such an analysis was run for 100,000 Markov chain Monte Carlo (MCMC) generations with a burn-in of 0.1 and a random seed of 123. The ML phylogenetic tree, including species delimitation results, was finally visualized using the Interactive Tree of Life (iTOL) v.5 tool (Letunic and Bork 2024).

### Phylogenetic analyses

**Fig. 2:**
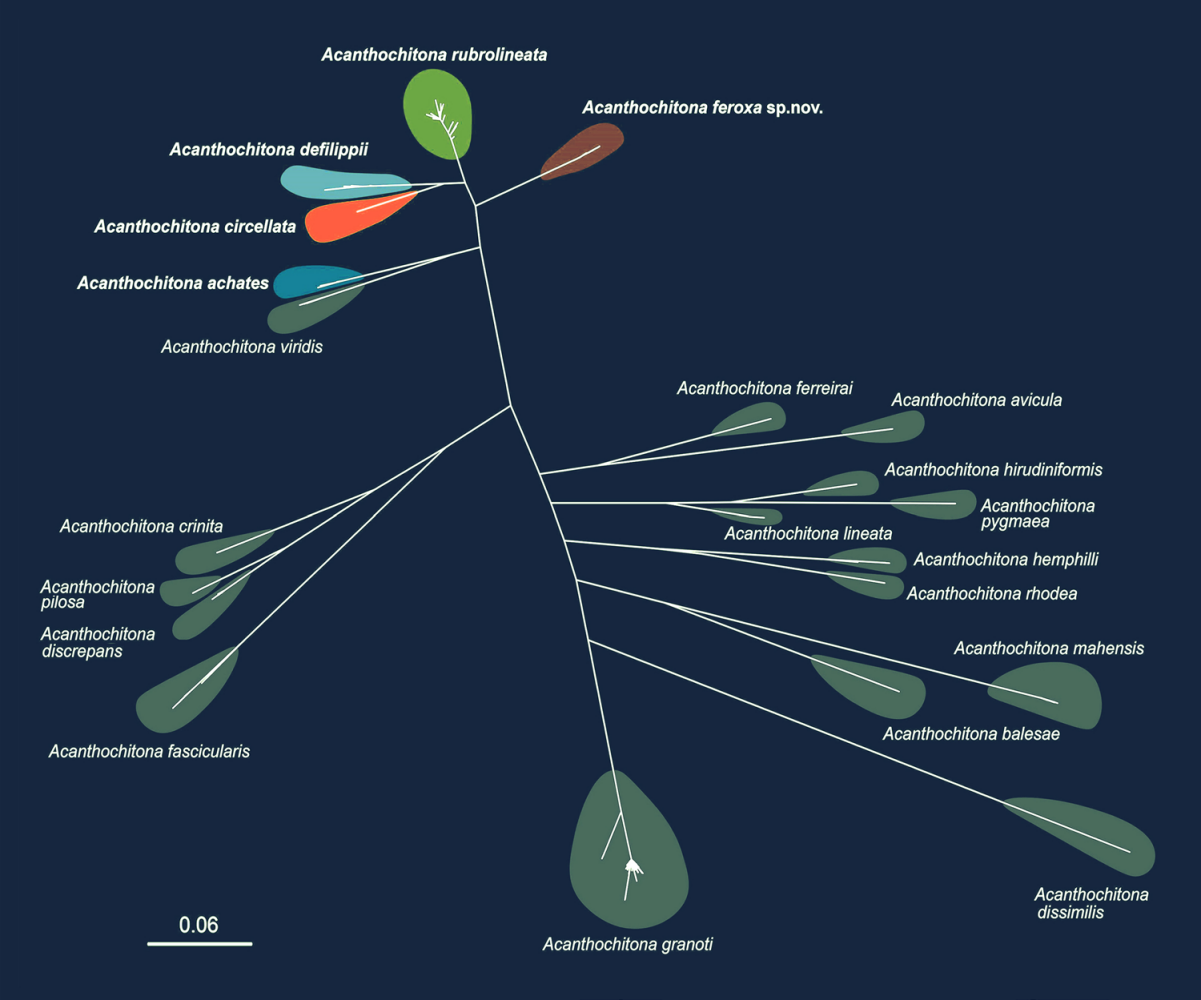
Unrooted phylogenetic tree illustrating the relationships among 21 *Acanthochitona* species, constructed using their mitochondrial *CO1* sequences

A phylogenetic analysis of the *CO1* sequences from 21 *Acanthochitona* species, including those retrieved from the NCBI and obtained in the present study, was conducted by aligning the 581 *CO1* sequences (Table 4; Fig. 2). The unrooted phylogenetic tree was reconstructed using the ML method through the IQ-TREE web server (http://iqtree.cibiv.univie.ac.at; Trifinopoulos et al. 2016). The best substitution model, HKY + F + I, was identified utilizing the model selection feature of IQ-TREE. The tree was generated with 1000 ultrafast bootstrap replicates.

**Table 4:** GenBank accession numbers and references for the *CO1* sequences of 21 *Acanthochitona* species used to construct the unrooted maximum likelihood phylogenetic tree

No	Species	GenBank accession number(s)	Reference(s)
1	*Acanthochitona achates*	PQ301026	This study
		MG679991–MG680001	Eernisse et al. 2018
		HM180416	Kim et al. 2012
2	*Acanthochitona avicula*	KJ574093	Irisarri et al. 2014
		NC047426	Irisarri et al. 2020
3	*Acanthochitona balesae*	KF184979	Ríos et al. 2014
4	*Acanthochitona circellata*	PQ301027	This study
5	*Acanthochitona crinita*	OR526579, OR145403–OR145408	Vončina et al. 2023
6	*Acanthochitona defilippii*	PQ651609–PQ651619	This study
		HM180425, HM180426	Kim et al. 2012
		OL877115, OL877116	Unpublished
		MG680031–MG680034	Eernisse et al. 2018
		LC214417	Owada 2023
7	*Acanthochitona discrepans*	OR145401, OR45402, OR526580–OR526594	Vončina et al. 2023
		PP748476–PP748480	Strack and Leotta 2024
8	*Acanthochitona dissimilis*	LC718174	Owada 2023
9	*Acanthochitona fascicularis*	PP945800	Unpublished
		OR145409, OR526595–OR526598	Vončina et al. 2023
10	*Acanthochitona ferreirai*	KC669568–KC669570	Ríos et al. 2014
		MK016365–MK016367	Ibáñez 2017
11	*Acanthochitona feroxa* sp. nov	PQ651620–PQ651679	This study
		HM180423	Kim et al. 2012
12	*Acanthochitona garnoti*	KP202499–KP202685	Wright et al. 2015
13	*Acanthochitona hemphilli*	KF184977, KF184978	Ríos et al. 2014
		KC669565–KC669567	Ríos et al. 2014
14	*Acanthochitona hirudiniformis*	MK016368–MK016370	Ibáñez 2017
15	*Acanthochitona lineata*	KF184980, KF184981	Ríos et al. 2014
16	*Acanthochitona mahensis*	OQ544537–OQ544553	Unpublished
		OM758209	Alnashiri et al. 2023
		NC082305	Unpublished
17	*Acanthochitona pilosa*	PP74848466–PP748481	Strack and Leotta 2024
18	*Acanthochitona pygmaea*	KF184982	Ríos et al. 2014
19	*Acanthochitona rhodea*	KC669564	Ríos et al. 2014
20	*Acanthochitona rubrolineata*	PQ775055–PQ775088	This study
		MN205753–MN205930	Ni et al. 2020
21	*Acanthochitona viridis*	MG680051–MG680051	Eernisse et al. 2018

To construct a mitochondrial genome alignment set representing the Polyplacophora, 23 sequences retrieved from the GenBank, including 19, one, and three species of Chitonida, Callochitonida, and Lepidopleurida, respectively (Table 5), were combined with those generated during this study. Additionally, five species of Caudofoveata: *Falcidens acutargatus*, *F*. *halanychi*, *Chaetoderma nitidulum*, *Scutopus ventrolineatus*, and *S*. *robustus* were included as outgroups. The PCG sequences were aligned utilizing the BioEdit software (Hall 1999) based on the ClustalW method (Thompson et al. 1994). Poorly aligned sites were removed, and all 13 PCG sequences were concatenated using the Gblocks 0.91b tool (Castresana 2000). A phylogenetic tree was reconstructed employing the ML method through the IQ-TREE web server. The best substitution model, GTR + F + I + G4, was selected by utilizing the ModelFinder tool (Kalyaanamoorthy et al. 2017). The phylogenetic tree was finally viewed, edited, and visualized using the Figtree 1.4.4 software (Rambaut 2018). A BI phylogenetic tree was constructed employing the MrBayes v3.2.7 program (Ronquist et al. 2012). The analysis was run with 4 MCMC chains for 10 million generations, with sampling at every 1000 generations. The initial 25% of the sampled trees were discarded as burn-in. To ensure the selection of an appropriate model and partitioning strategy applicable to the ML analysis, the concatenated dataset of the 13 PCGs was partitioned based on gene and codon position (1st, 2nd, and 3rd). The best-fitting nucleotide substitution model for each partition was selected using ModelFinder in IQ-TREE2.

**Table 5:** Classification, species names, mitochondrial genome sizes, GenBank accession numbers, and references for the 28 polyplacophoran and 5 caudofoveatan species (outgroups) used for ML tree reconstruction

Classification		Species	Size (bp)	GenBank accession number(s)	References
Polyplacophora	Acanthochitonidae	*Acanthochitona achates*	15,006	PQ301026	This study
		*Acanthochitona avicula*	15,203	NC047426	Irisarri et al. 2020
		*Acanthochitona circellata*	14,998	PQ301027	This study
		*Acanthochitona defilippii*	14,999	PP419021	Kim et al. 2024
		*Acanthochitona feroxa* sp. nov	14,991	OR757114	This study
		*Acanthochitona rubrolineata*	14,986	PQ301028	This study
	Callochitonidae	*Callochiton steinenii*	11,923	MN864061	Irisarri et al. 2020
	Chaetopleuridae	*Chaetopleura apiculate*	15,108	KY824658	Guerra et al. 2018
	Chitonidae	*Acanthopleura echinata*	14,848	MN864062	Irisarri et al. 2020
		*Acanthopleura loochooana*	15,366	NC068064	Unpublished
		*Acanthopleura vailanti*	15,271	OQ355692	Alnashiri et al. 2024
		*Chiton albolineatus*	14,936	NC047425	Irisarri et al. 2020
		*Sypharochiton pelliserpentis*	15,048	NC024174	Veale et al. 2016
		*Sypharochiton sinclairi*	15,208	NC024173	Veale et al. 2016
		*Tonicia forbesii*	14,569	MN864054	Irisarri et al. 2020
	Ischnochitonidae	*Lepidozona coreanica*	16,572	NC046935	Sun et al. 2023
	Lepidochitonidae	*Cyanoplax caverna*	15,141	NC026848	Irisarri et al. 2014
		*Nuttallina californica*	15,604	NC026849	Irisarri et al. 2014
	Leptochitonidae	*Hanleyella oldroydi*	15,692	NC047423	Irisarri et al. 2020
		*Leptochiton nexus*	15,488	NC047422	Irisarri et al. 2020
	Mopaliidae	*Cryptochiton stelleri*	15,082	NC026850	Irisarri et al. 2014
		*Dendrochiton gothicus*	15,288	NC047424	Irisarri et al. 2020
		*Katharina tunicate*	15,288	NC047424	Irisarri et al. 2020
		*Mopalia retifera*	14,984	NC068065	Unpublished
		*Nuttallochiton mirandus*	12,511	MN864053	Irisarri et al. 2020
		*Plaxiphora albida*	15,078	MN864050, MN864052	Irisarri et al. 2020
		*Tonicina zschaui*	12,914	MN864051	Irisarri et al. 2020
	Nierstraszellidae	*Nierstraszella lineata*	15,765	NC047421	Irisarri et al. 2020
Caudofoveata (Outgroup)		*Chaetoderma nitidulum*	21,008	NC013846	Unpublished
		*Falcidens acutargatus*	14,209	NC037876	Mikkelsen et al. 2018
		*Falcidens halanychi*	14,508	NC037877	Mikkelsen et al. 2018
		*Scutopus robustus*	14,515	NC038081	Mikkelsen et al. 2018
		*Scutopus ventrolineatus*	14,662	NC025284	Osca et al. 2014

### Description of *Acanthochitona* sp. nov.

The 59 specimens for the *A*. sp. nov. type series were deposited under voucher numbers LEGOM040557–LEGOM040615. The chitons were observed under a M205 C stereo microscope (Leica Camera AG, Wetzlar, Germany). The images were captured using a mounted MC190 HD camera (Leica) and presented using the focus-stacking software, Helicon Focus program v8.2.2 (https://www.heliconsoft.com/software-downloads/). Some individuals were dissected to examine their valves, girdle, and radulae. Their microstructural features were captured with a SU8220 field emission scanning electron microscope (FE-SEM) (Hitachi, Tokyo, Japan). The final image panels were prepared using photoshop CS6 (Adobe Inc., CA, USA).

### Divergence time estimation

Divergence times for the 28 polyplacophoran species were estimated based on the mitochondrial genome sequences using the BEAST 2.7.7 cross-platform program (Bouckaert et al. 2014). A relaxed molecular clock model under a calibrated Yule tree was applied, with four calibration points derived from fossil records. The first point, *Echinochiton dufoei*, from the Ordovician Period (ca. 449.5–549 Ma), represents the divergence between Aplacophora and Polyplacophora (Pojeta et al. 2003). The second point constrains the origin of Lepidopleurida to 201.3–359 Ma, based on the *Leptochiton* spp. fossils from the Upper Triassic (Laghi 2005; Sirenko 2013). The third point, for Chitonida, was set at 174–359 Ma, based on Jurassic fossils, such as *Allochiton* and *Heterochiton* (Fucini 1912), and *Ischnochiton marloffsteinensis* (Fiedel and Keupp 1988). The final point, 33.9–359 Ma, marks the divergence of the crown-group Acanthochitonina, supported by the *Plaxiphora* spp. and *Acanthochitona* spp. fossils (Puchalski et al. 2008). The analysis was executed for 1,000,000 MCMC iterations and sampled every 1000 iterations after discarding the initial 10% as burn-in. The “monophyly” option in the BEAUti program was chosen, and GTR was used as the best-fit substitution model. The TreeAnnotator 2.7.7 program was employed to produce a phylogenetic tree with maximum clade credibility, and the node heights were set as median heights to annotate the tree. The output tree was visualized using FigTree 1.4.4 (Rambaut 2018).

## Results

### Mitochondrial genomes of the five *Acanthochitona* species

We completely sequenced the mitochondrial genomes of the five *Acanthochitona* species, producing fragments with lengths of 15,006 bp for *A*. *achates*, 14,998 bp for *A*. *circellata*, 14,999 bp for *A*. *defilippii*, 14,986 bp for *A*. *rubrolineata*, and 14,991 bp for *A*. *feroxa* sp. nov. (Fig. 1). The nucleotide compositions were 29.79–31.82% for A, 39.02–39.87% for T, 16.69–18.53% for G, and 11.62–12.66% for C. The genomes exhibited a strong A/T bias with an AT content of 68.81–71.68%. The longest sequence was of *A*. *achates*, whereas the shortest was of *A*. *rubrolineata*, with a difference of 20 bp between the two. All genomes were circular, double-stranded DNA molecules containing the 37 typical coding genes: 13 PCGs, 22 tRNA genes, and two rRNA genes. Of them, 16 genes, including seven PCGs (*ATP6*, *ATP8*, *COX1*, *COX2*, *COX3*, *ND2*, and *ND3*) and nine tRNA genes (*trnA*, *trnD*, *trnI*, *trnK*, *trnN*, *trnP*, *trnR*, *trnS1*, and *trnT*), were located on the heavy strand. The remaining 21, consisting of 6 PCGs (*CytB*, *ND1*, *ND4*, *ND4L*, *ND5*, and *ND6*), 13 tRNA genes (*trnC*, *trnE*, *trnF*, *trnG*, *trnH*, *trnL1*, *trnL2*, *trnM*, *trnQ*, *trnS2*, *trnV*, *trnW*, and *trnY*), and two rRNA genes (*rrnS* and *rrnL*), were located on the light strand.

The PCGs were between 11,172 and 11,223 bp in length. The longest PCG was *ND5*, at 1713 bp, while the shortest was *ATP8*, at 162 bp. ATG was the common start codon in most PCGs, with a few exceptions: *ND4* of *A*. *achates* and *ND6* of *A*. *circellata* with ATT, and *ND4* of *A*. *feroxa* sp. nov. with GTG. Both these codons have been identified as alternative start codons in the mitochondrial systems of other animals (Boore and Brown 1994; Montoya et al. 1981). Most of the PCGs terminated with the stop codons TAG or TAA, and some others with an incomplete stop codon, containing only T, as observed in the *ND6*s of *A*. *defilippii* and *A*. *achates*. This T is typically added during transcription to form a complete stop codon, T(AA) (Ojala et al. 1981). All tRNAs, except *trnS2*, possessed a typical cloverleaf structure, 61–68 bp in length; *trnS2* lacked the D-arm, a characteristic feature of the phylum Mollusca. Watson–Crick base pairs (A–T and G–C) were frequent in the stem regions; G–U wobble and mismatched pairs were also found.

The 13 PCGs of all *Acanthochitona* species comprised 3717 codons in *A*. *achates*, 3719 in *A*. *circellata*, 3721 in *A*. *defilippii,* 3724 in *A*. *rubrolineata,* and 3727 in *A*. *feroxa* sp. nov., with relatively similar amino acid compositions (Supplementary Fig. 1). Among the 22 amino acids, Ser2 comprised the highest proportion, at the same levels across all five species. A comparison of the relative synonymous codon usages (RSCUs) of the five *Acanthochitona* species revealed that AGA (*Ser1*), UCU (*Ser2*), and UUA (*Leu2*) were the most frequent, while CUG (*Leu1*), ACG (*Thr*), and GTC (*Val*) were the least.

### Mitochondrial genome-based phylogeny of Polyplacophora

**Fig. 3:**
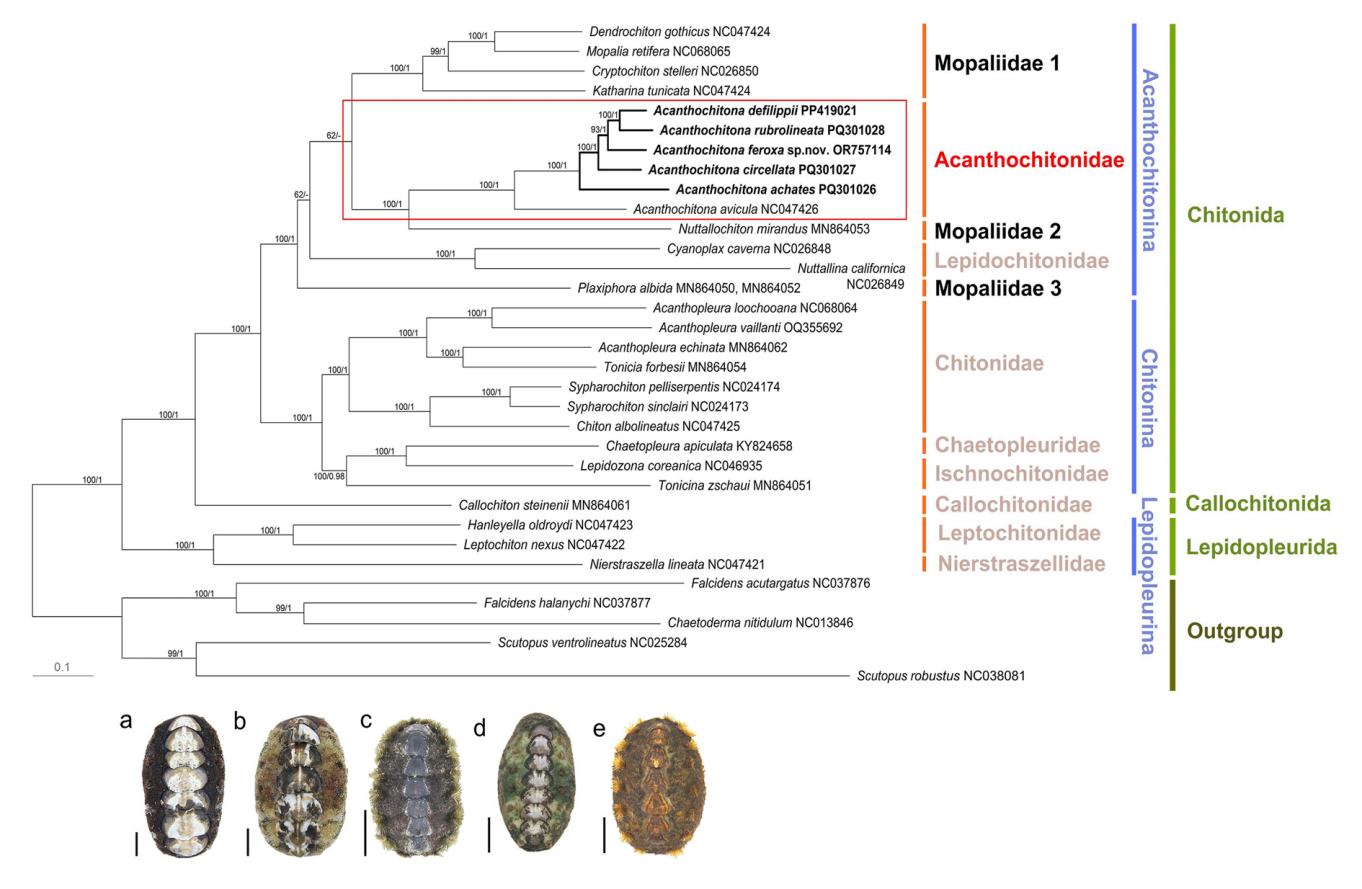
Maximum likelihood tree reconstructed based on the concatenated nucleotide sequences of 13 mitochondrial PCGs from 28 chitonid species. The phylogenetic dataset consisted of Chitonida (24), Callochitonida (1), and Lepidopleurida (3). The focal species are indicated in boldface, and their dorsal views are shown. These include **a** *A*. *achates*, **b** *A*. *circellata*, **c** *A*. *defilippii*, **d** *A*. *rubrolineata*, and **e** *A*. *feroxa* sp. nov. (scale bars = 5 mm). The outgroup consisted of five Caudofoveata species: *F*. *acutargatus*, *F*. *halanychi*, *C*. *nitidulum*, *S*. *ventrolineatus*, and *S*. *robustus*. The GenBank accession numbers are included next to or below the species names. The numbers above the branches indicate the ML bootstrap support values and Bayesian posterior probabilities (BP/BPPBI). The *Acanthochitona* species included in this study are highlighted in red boxes

The 13 PCGs within the mitochondrial genomes of the 28 polyplacophoran species, including 24, one, and three species belonging to the orders Chitonida, Callochitonida, and Lepidopleurida, respectively, were used to construct a concatenated sequence alignment dataset (Fig. 3). Additionally, five Caudofoveata species: *F*. *acutargatus*, *F*. *halanychi*, *C*. *nitidulum*, *S*. *ventrolineatus*, and *S*. *robustus* served as the outgroups.

The class Polyplacophora formed a monophyletic group with a bootstrap value (BP) of 100 and was subdivided into three orders: Chitonida, Callochitonida, and Lepidopleurida, each exhibiting monophyly. Most species within Chitonida were divided between two main groups representing the suborders Acanthochitonina and Chitonina. Among the nine polyplacophoran families included, seven were monophyletic with bootstrap values ranging from 70 to 100, whereas Mopaliidae and Ischnochitonidae did not form any monophyletic group. The five *Acanthochitona* species clustered with *A*. *avicula* belonging to the family Acanthochitonidae and the suborder Acanthochitonina, forming a highly supported (BP 100) branch. *Nuttallochiton mirandus*, a Mopaliidae member, appeared as a sister taxon to the six *Acanthochitona* species (BP 100). Interestingly, while entirely within Acanthochitonina, Mopaliidae is not supported as monophyletic, but is instead divided into three distinct genetic lineages. Additionally, *Lepidozona coreanica*, a member of the Ischnochitonidae family, was grouped along with *Chaetopleura apiculata*, belonging to Chaetopleuridae, a sister group of Ischnochitonidae, making Ischnochitonidae polyphyletic. The BI tree demonstrated high node support with most posterior probabilities close to 1 and a topology largely consistent with the ML tree, although Leptochitonidae was positioned differently between the two trees (Supplementary Figs. S2, S3).

### Mitochondrial gene arrangements within Polyplacophora

The mitochondrial gene order exhibited a marked conservation among polyplacophoran species (Supplementary Fig. S4), being generally consistent with that found in *Chaetopleura apiculata*, a proposed model for the ancestral gene order (Guerra et al. 2018; Irisarri et al. 2020). This gene order was followed in all six *Acanthochitona* species within Acanthochitonidae (*A*. *achates*, *A*. *circellata*, *A*. *defilippii*, *A*. *rubrolineata*, *A*. *feroxa* sp. nov., and *A*. *avicula*), three species within Mopaliidae (*Dendrochiton gothicus*, *Mopalia retifera*, and *Plaxiphora albida*), five species within Chitonidae (*Acanthopleura echinata*, *A*. *loochooana*, *A*. *vaillantii*, *Tonicia forbesii*, and *Chiton albolineatus*), two within Ischnochitonidae (*Lepidozona coreanica* and *Tonicina zschaui*), and *Callochiton steinenii* within Callochitonidae. However, some exceptions were also detected. Within the order Lepidopleurida, the families Leptochitonidae and Niestraszellidae exhibited significant gene rearrangements. The *trnF* was inverted in *Nierstraszella lineata* (Niestraszellidae), while *trnE* was duplicated in *Hanleyella oldroydi* and *Leptochiton nexus* (Leptochitonidae). Inversions of *trnV* and *trnW* were also detected in *Sypharochiton pelliserpentis* and *S*. *sinclairi* (order: Chitonida and suborder: Chitonina). Several rearrangements were also observed in the suborder Acanthochitonina, including an inversion of the *trnM*–*trnC*–*trnY*–*trnW*–*trnQ*–*trnG*–*trnE* cluster in *Cyanoplax caverna* and *Nuttallina californica* (Leptochitonidae) and of *trnH* in *Cryptochiton stelleri* (Mopaliidae). Additionally, *Katharina tunicata* (Mopaliidae) showed a translocation of *trnD* from after *CO2* to between *CO1* and *CO2*.

### CO1 gene diversity among the three *Acanthochitona* species

We used the *CO1* sequences of 295 individuals to examine the genetic divergence among three *Acanthochitona* species: *A*. *defilippii*, *A*. *rubrolineata*, and *A*. *feroxa* sp. nov. Of these, 105 individuals were collected from the five intertidal regions of South Korea (Table 2). We PCR-amplified the *CO1* barcoding region, which yielded 510 bp long amplicons that were subsequently sequenced. In addition, we retrieved 190 previously published *CO1* sequences (all 510 bp long) from the NCBI database, including nine from *A*. *defilippii* (Kim et al. 2012) and 180 from *A*. *rubrolineata* (Ni et al. 2020). We aligned the nucleotide sequences of the 97 *CO1* haplotypes from 295 individuals, identifying 97 *CO1* haplotype sequences (Table 3): 13 from *A*. *defilippii*, 15 from *A*. *feroxa* sp. nov., and 69 from *A*. *rubrolineata*. Genetic diversity analysis of these haplotypes revealed an average haplotype diversity (*h*) of 0.773 and nucleotide diversity (*π*) of 0.01173 (Table 3). The highest *h* (1) was observed in Kinaisemen (KI) and Nomomachi (NM), Japan, while the maximal *π* (0.04363) was found in Yeosu (YS), South Korea. The most abundant haplotype comprising 48 individuals was also found in YS.

### Molecular identification of three *Acanthochitona* species

**Fig. 4:**
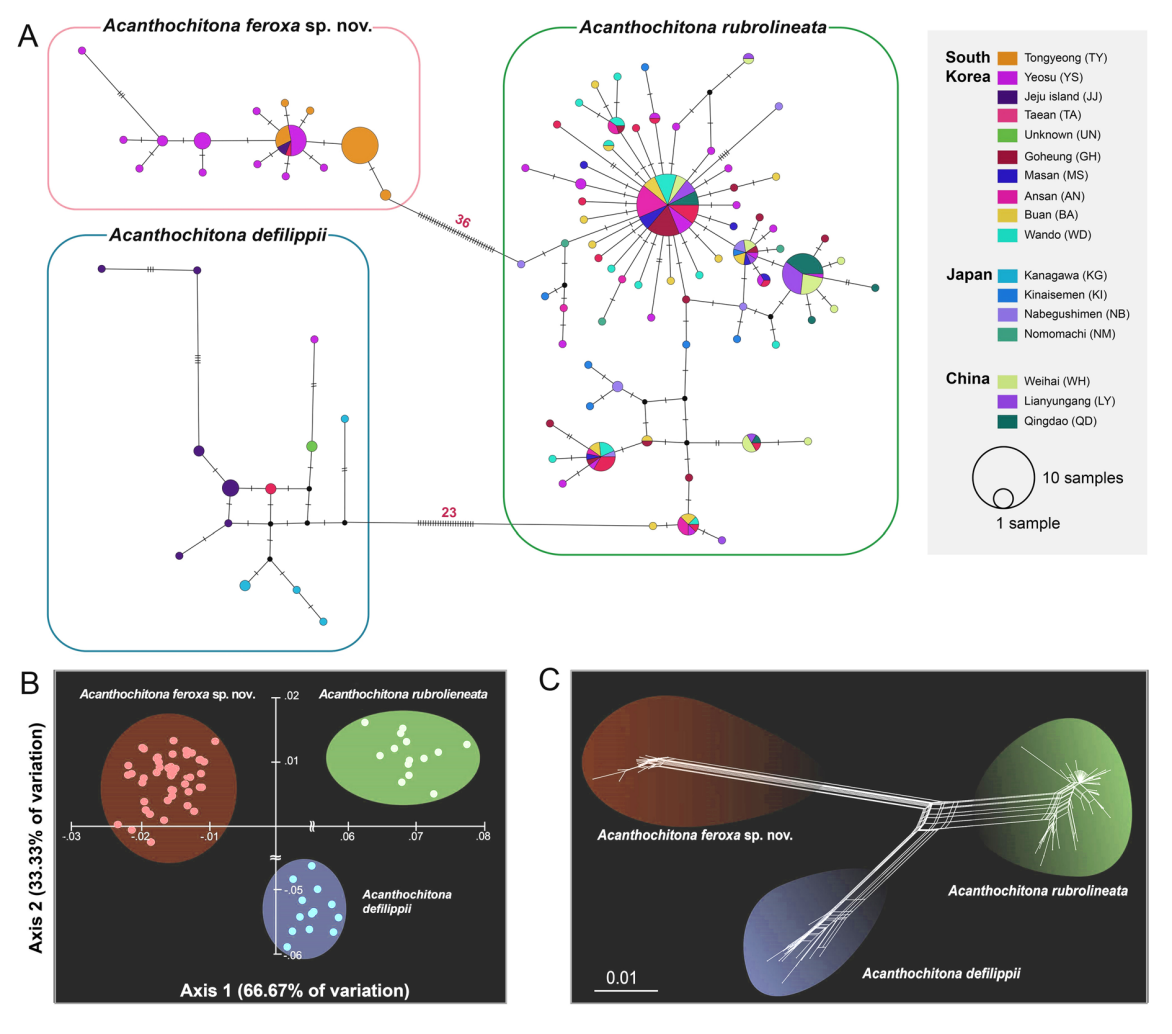
TCS haplotype network, PCoA plot, and phylogenetic network, constructed based on 97 *CO1* haplotypes from 295 individuals belonging to three *Acanthochitona* species: *A*. *defilippii*, *A*. *rubrolineata*, and *A*. *feroxa* sp. nov., illustrate their genetic distinctiveness. **A** The TCS haplotype network indicates three distinct genetic clusters corresponding to *A*. *defilippii*, *A*. *rubrolineata*, and *A*. *feroxa* sp. nov. The circle size is proportional to the haplotype frequency. The small black circles represent the hypothetical haplotypes. The crossbars on the lines indicate the number of nucleotide substitutions. **B** The 2-dimensional PCoA plot demonstrates three distinct genetic groups corresponding to *A*. *defilippii*, *A*. *rubrolineata*, and *A*. *feroxa* sp. nov. The scores of the two axes (Axis 1 = 66.67%, and Axis 2 = 33.33%) are included. **C** The phylogenetic network tree, reconstructed using the neighbor-net algorithm without an outgroup, also shows three different genetic lineages

The TCS network divided the 97 *CO1* haplotypes into three distinct groups (Fig. 4A). Among them, 28 belonged to two groups, containing 13 or 15 haplotypes detected in *A*. *defilippii* and in *A*. *feroxa* sp. nov., respectively (Fig. 4A). The remaining 69 were identified in *A*. *rubrolineata*. Additionally, *A*. *defilippii* was separated from *A*. *rubrolineata* by 23 step-wise mutations, while *A*. *feroxa* sp. nov. was separated from *A*. *rubrolineata* by 36. Thus, *A*. *rubrolineata* is genetically closer to *A*. *defilippii* than to *A*. *feroxa* sp. nov., and *A*. *defilippii* was not directly related to *A*. *feroxa* sp. nov., indicating a distinct genetic differentiation among all three species. Consistent with this finding, the PCoA plot (Fig. 4B) and unrooted phylogenetic network (Fig. 4C) further supported the presence of three distinct groups corresponding to *A*. *defilippii*, *A*. *rubrolineata*, and *A*. *feroxa* sp. nov.

Using ABGD, the pairwise genetic divergences were distributed (Supplementary Fig. S5A), ranked pairwise differences were identified (Supplementary Fig. S5B), and *CO1-*based automatic partition analyses were conducted (Supplementary Fig. S5C), which confirmed the two distinct barcoding gaps, representing intraspecific and interspecific variations, respectively, strongly supporting division into three species. In addition, species delimitation analyses using ASAP and poisson tree process (PTP) also supported this categorization. The ASAP analysis consistently identified three optimal partitions with the lowest ASAP scores (Supplementary Fig. S5D), while the ML phylogenetic tree-based PTP delineated three putative species with robust support values (Supplementary Fig. S5E). These complementary results obtained from multiple methods provide robust evidence for the presence of three distinct species within the analyzed taxa.

### Morphological differences among the five *Acanthochitona* species

We compared the morphological characteristics of the five *Acanthochitona* species (Fig. 6) and described a new *Acanthochitona* species, *A*. *feroxa* sp. nov., Kim and Hwang, 2024, from South Korea. The morphological characteristics observed under a M205 light microscope and a SU8220 FE-SEM have been described in detail.

Class: Polyplacophora Gray, 1821.

Order: Chitonida Thiele, 1909.

Family: Acanthochitonidae Pilsbry, 1893.

Genus: *Acanthochitona* J. E. Gray, 1821.

***Acanthochitona feroxa* sp. nov.** (Fig. 3e)

**Etymology.** The species-group name *feroxa* is derived from the Latin *ferox*, meaning “fierce” or “bristling”, reflecting the characteristic sharp tufts of this species.

**Type locality.** 34°52′11’’N, 128°24′12’’E, Tongyeong-si, South Korea, collected by I Hyang Kim, 3 May 2023.

**Type material.** [Holotype] South Korea: one specimen, Gyeongsangnam-do, Tongyeong-si, Gwangdo-myeon; [Paratype] South Korea: 32 specimens, Gyeongsangnam-do, Tongyeong-si; 25 specimens, Jeollanam-do, Yeosu-si, Hwayang-myeon; two specimens, Jeju-island, Seogwipo-si.

**Habitat.** Under stones in lower intertidal zones with muddy habitats.

**Measurements.** Length: 21 mm, width: 13 mm.

**ZooBank registration number.** urn:lsid:zoobank.org:act:9B9BA6AC-9837-494A-B4FC-8D48A901899E.

**Description.** Body oval-shaped, with small, low, round-backed valves. Color of the tegmentum gray, black, or light brown; articulamentum and hypostracum bluish white.

Head valve semicircular in shape; pustules arranged in irregular rows, largest near the margin, progressively smaller toward the apex; anterior margin rounded at the anterior end of jugum; posterior margin nearly straight or slightly convex. Articulamentum white to light blue, well developed, moderately thick; insertion plate symmetric and short, with five slits.

Intermediate valves are roughly trapezoidal in shape; jugum area is smooth and wide; anterior margin is convex; posterior margin has pustules that are thin and long; lateral areas are hardly separable. Articulamentum is white to light blue; anterior margin is round; there is one slit on each side of the insertion plate.

Tail valve small and circular; mucro slightly behind center, with scattered pustules in the postmucronal area; posterior margin somewhat notched. Articulamentum white to light blue; insertion plate symmetric, long, extends upward; roughly truncated in the tail valve with five slits.

Girdle wide, ranging in color from dark brown to green and white; covered with two distinctly sized spicules: small, smooth, rounded dorsal spicules and long, thick, pointed dorsal spicules. Nine pairs of sutural tufts are prominent; sutural tuft needles are straight, smooth, and sharp, thinner than the dorsal needles.

Pustules oval to elongated in shape; densely packed; surface of pustules flat or slightly concave; usually in a parallel arrangement; each pustule with one central macraesthete pore.

Ventral spicules are smooth, short, thick, and rounded. Gills extend on each side from valves III to VII, composed of 22 ctenidia on each side. Gill arrangement is adanal and holobrachial.

Radula consists of symmetrical rows. Central tooth with a round upper part and concave sides; the basal part is narrow. Centro-lateral tooth is rather thin, antero-dorsal corner is slightly notched. Major lateral tooth head tricuspid; cusps sharply pointed; central cusp slightly longer than the others. Major uncinal tooth long, wide S spoon-like in shape on the upper side. Marginal tooth flattened, slightly dented on the central part.

**Distribution.** West Sea and South Sea coasts of South Korea.

**Fig. 5:**
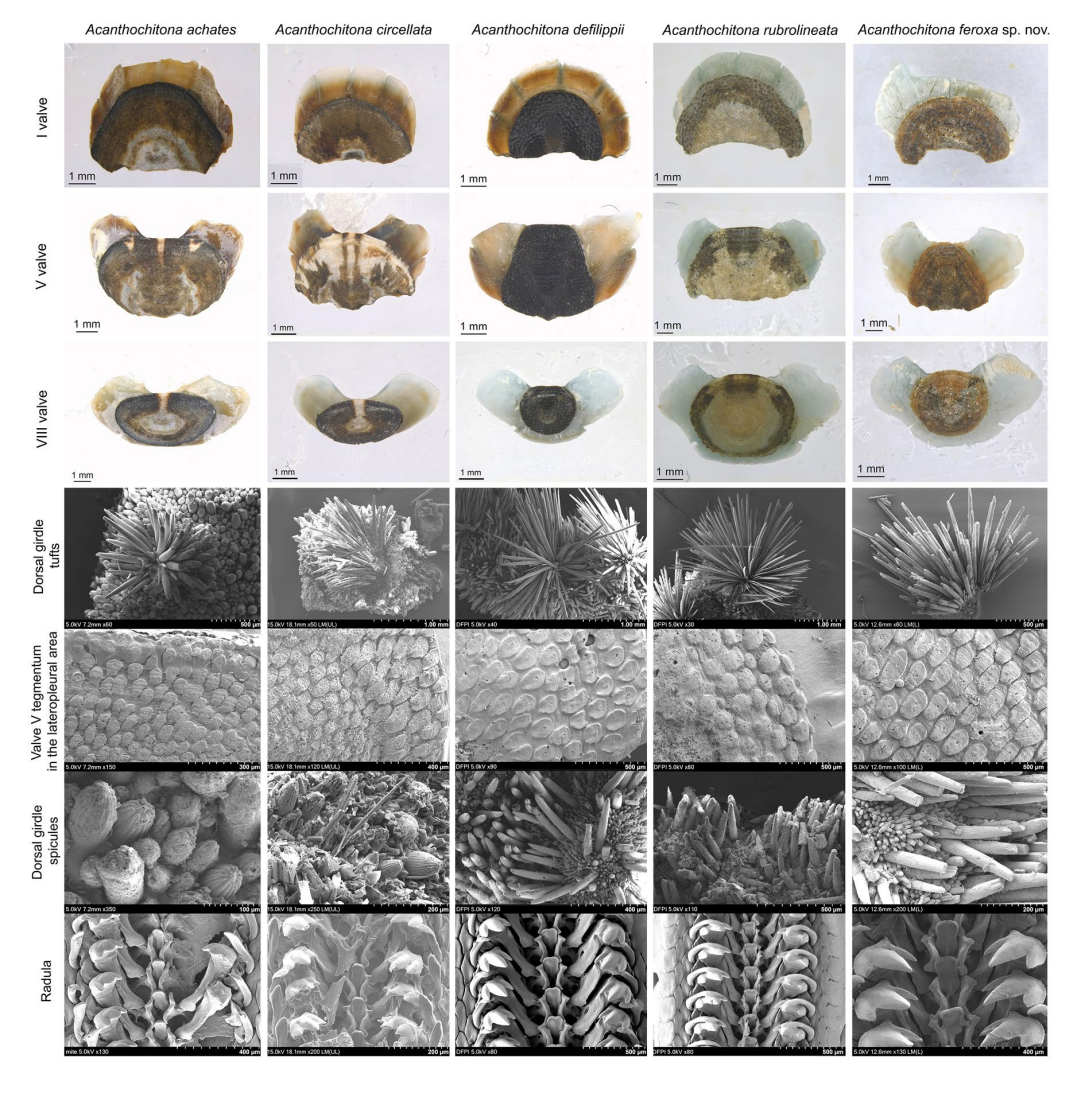
Morphological comparison of five *Acanthochitona* species. Valves I, V, and VIII were photographed under a light microscope mounted with a Leica MC190 HD camera and processed using Helicon Focus v8.2.2. Dorsal girdle tufts, the lateropleural area of valve V tegmentum, dorsal girdle spicules, and radula were imaged using a Hitachi SU8220 field emission scanning electron microscope

**Remarks.** The specimens examined exhibited a variation in the number of gills, with some individuals possessing 19 and others 21, which may be related to ontogenetic differences. Additionally, the specimens, found in mud-rich habitats, showed significant wear on the girdle setae and shell plates, likely due to the environmental conditions. We compared the morphological characteristics of *A*. *feroxa* sp. nov. with those of the four other *Acanthochitona* species: *A*. *achates*, *A*. *circellata*, *A*. *defilippii*, and *A*. *rubrolineata*. *A*. *feroxa* sp. nov. possessed a smaller and more circular tail valve and long, thin dorsal girdle spicules compared to *A*. *achates* and *A*. *circellata* (Fig. 5). Additionally, in comparison with *A*. *rubrolineata*, *A*. *feroxa* sp. nov. exhibited a narrower, trapezoidal intermediate valve without a central line and elongated granules in the tegmentum of the fifth valve. Compared with the morphologically similar *A*. *defilippii*, *A*. *feroxa* sp. nov. was distinguished by rounded dorsal spicules and a straight, rather than notched, apical margin of its upper central tooth (Fig. 5). Additionally, compared to *Acanthochitona viridis*, which is closely related to the other four *Acanthochitona* species, *A*. *feroxa* sp. nov. does not have a thin, mid-plate white line, and the plate color is almost blue-less.

Based on these morphological differences, we constructed a user-friendly identification guide for the five valid species of *Acanthochitona* examined in this study (Supplementary Fig. 6). One of the *Acanthochitona feroxa* sp. nov. specimens has been deposited in the Honam National Institute of Biological Resources, Mokpo, South Korea (HNIBR No. HNIBRIV12838).

### Divergence times of the major polyplacophoran groups

**Fig. 6:**
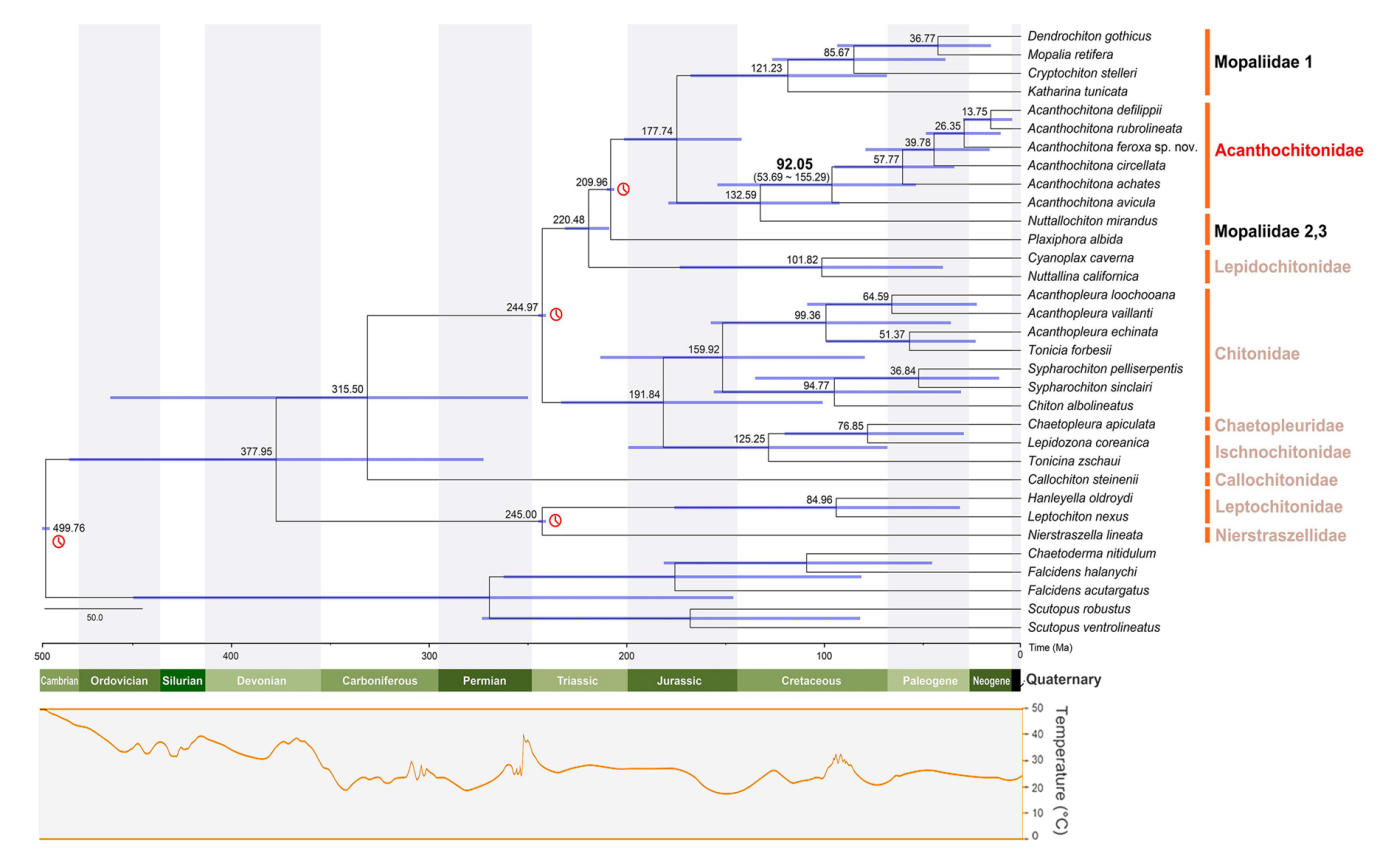
Divergence times were estimated employing the 13 PCGs belonging to 28 polyplacophoran species. A time-calibrated Bayesian tree was reconstructed using BEAST, incorporating calibration points (red clock symbols) which were based on fossil records. The first point, representing the divergence between Aplacophora and Polyplacophora, was based on *Echinochiton dufoei* fossils from the Ordovician period (ca. 449.5–549 Ma; Pojeta et al. 2003). The second point constrains the origin of Lepidopleurida to 201.3–359 Ma based on the fossils of *Leptochiton* spp. from the Upper Triassic (Laghi 2005; Sirenko 2013). The third point, constraining the divergence of Chitonida, was set at 174–359 Ma, based on the Jurassic period fossils of genera, such as *Allochiton* (Fucini 1912), *Heterochiton* (Fucini 1912), and *Ischnochiton marloffsteinensis* (Fiedel and Keupp 1988). The final point, 33.9–359 Ma, was supported by *Plaxiphora* spp. and *Acanthochitona* spp. fossils (Puchalski et al. 2008). The image was edited using Adobe Illustrator v25.2 (Adobe Inc., CA, USA)

An analysis of the divergence times suggested that the first diversification event within the crown group of Polyplacophora occurred 377.95 Ma (95% highest posterior density interval [HPD]: 275.07–487.17) during the Carboniferous period (Fig. 6). The Callochitonidae and the remaining Chitonida split 315.50 Ma (95% HPD: 252.24–466.18) during the Carboniferous. The ages of Chitonida (excluding Callochiton) and Lepidopleurida were estimated to be 244.97 Ma (95% HPD: 243.12–246.98) and 245.00 Ma (95% HPD: 243.12–246.99), respectively, placing them in the Triassic period. Within Chitonina, the divergence between Chaetopleuridae and Ischnochitonidae from Chitonidae was estimated to have occurred ~ 191.84 Ma. Chaetopleuridae and Ischnochitonidae were suggested to have split ~ 125.25 Ma, and the first major one in Chitonidae was estimated to have occurred at ~ 159.92 Ma, placing it in the Jurassic period. The earliest divergence in Acanthochitonina was estimated at ~ 209.96 Ma, with a further one within Lepidochitonidae at 142.95 Ma. The clade representing most Mopaliidae members was calculated to have diverged 101.82 Ma. For Acanthochitonidae, lineage divergence appears to have followed the separation of *N*. *mirandus* (Mopaliidae) 132.59 Ma, with a subsequent split within *Acanthochitona* estimated at 92.05 Ma (95% HPD: 53.69–155.29).

## Discussion

### Systematic and evolutionary insights into the genus *Acanthochitona*

*Acanthochitona* comprises polyplacophorans characterized by 18 tufts inhabiting diverse environments from intertidal zones to deep-sea ecosystems worldwide. These distinctive features are key to species identification, along with various other studied ones. Research on morphological differentiation began with Lyons (1988), who focused on the epidermal structure as well as the number and placement of sensory organs (aesthetes) in the *Acanthochitona* species occurring in the Caribbean and Central America. Subsequent studies by Watters (1990) and Ríos et al (2014) further elucidated the roles of epidermal, radular, and girdle features in identifying Caribbean *Acanthochitona* species. However, individual variability can complicate species identification based on morphological differences, due to which molecular analysis has emerged as an effective alternative tool. Particularly, mitochondrial genome analysis is useful in distinguishing closely related species due to its high variability and matrilineal inheritance patterns (Hebert et al. 2003; Rubinoff 2006). These molecular techniques have been proven potent in uncovering cryptic species within groups that exhibit morphological similarities (Choi et al. 2021a, b; Park et al. 2022).

Mitochondrial genome analysis helped identify a cryptic species within *A*. *defilippii* that is morphologically similar yet genetically distinct. Using the molecular species identification marker *CO1*, we performed TCS network, PCoA, and phylogenetic network analyses, and also examined the DNA barcoding gaps. These efforts distinguished three groups—*A*. *defilippii*, *A*. *rubrolineata*, and *A*. *feroxa* sp. nov.—suggesting them to be representative of distinct *Acanthochitona* species. Furthermore, FE-SEM revealed morphological variations, particularly in the dorsal spicules and radula. To date, this species has been collected and identified only in South Korea, suggesting the possibility of it being endemic.

We also characterized the mitochondrial genomes of five *Acanthochitona* species, providing foundational data critical for further research. Our analysis of mitochondrial gene rearrangements indicated that tRNA often acted as the most dynamic element. The commonly employed tandem duplication–random loss model (Boore 2000) accounted for the translocation of *trnD* in *K*. *tunicata*, as well as the duplication of *trnE* in *L*. *nexus* and *H*. *oldroydi*. This duplication occurs within a non-coding region, considered a hotspot for gene rearrangements (San Mauro et al. 2006). The inversions of tRNA genes in *C*. *caverna*, *N*. *californica*, *C*. *stelleri*, and *Sypharochiton* can be explained by alternative mechanisms, such as abnormal recombination via minicircles (Dowton and Campbell 2001). These can serve as valuable phylogenetic markers that can help clarify the taxonomy within various lineages.

### Phylogenetic relationships and divergence times in Polyplacophora

We reconstructed the polyplacophoran phylogeny using the nucleotide sequences of the 13 PCGs. The five *Acanthochitona* species analyzed clustered closely with *A*. *avicula*, forming a robust monophyletic group within the family Acanthochitonidae. Additionally, the newly described species, *A*. *feroxa* sp. nov., was identified as a distinct one, forming a separate lineage within the ML tree with a bootstrap value of 93. It was closely related to *A*. *defilippii* and *A*. *rubrolineata*. Most bootstrap values across the phylogenetic tree exceeded 60, indicating high topology confidence.

As seen in Fig. 3 and Supplementary Fig. S3, Acanthochitonidae (BPs = 100 and 1) formed a monophyletic group but clustered with Mopaliidae, which did not belong to a single lineage. Instead, Mopaliidae separated into three distinct lineages, consistent with previous molecular studies (Irisarri et al. 2014, 2020; Okusu et al. 2003). The first lineage, “Mopaliidae 1”, including *D*. *gothicus*, *M*. *retifera*, *C*. *stelleri*, and *K*. *tunicata*, appeared to be a sister group of the monophyletic clade (BP = 100, BPP = 1) of Acanthochitonidae + *Nuttallochiton mirandus* (Mopaliidae 2). Finally, the third lineage, “Mopaliidae 3”, represented by *P*. *albida*, occupied the basal position within the Acanthochitonina, aligning with its morphological resemblance to Acanthochitonidae in terms of aesthete structure (Vendrasco et al. 2008).

Such a polyphyletic pattern indicates that morphological traits traditionally associated with Mopaliidae—such as broad body outlines, corneous or setose girdles, and a posterior sinus in the tail valve—may have arisen independently through convergent evolution rather than a common ancestry (Sirenko 2006; Van Belle 1983). Although *Plaxiphora* and other mopaliid taxa are phylogenetically distant, they share a broad body outline and a girdle covered with corneous hairs or setae, traits most likely shaped by similar environmental pressures occurring in wave-exposed or sediment-rich habitats (Eernisse and Reynolds 1994; Vendrasco et al. 2008). Furthermore, the posterior valve sinus—often considered diagnostic of Mopaliidae—may instead reflect functional adaptations for respiration, substrate attachment, or defense (Boore and Brown 1994; Sirenko 2006; Van Belle 1983). These findings underscore the need for additional research, by combining molecular, morphological, and ecological data, to refine mopaliid taxonomy, elucidate the roles of convergent evolution in chiton diversification, and illuminate the significance of these traits in adaptation to various marine environments (Giribet et al. 2006).

Further emphasizing the complexity among mopaliid relationships, *N*. *mirandus* is strongly supported to be closely related to Acanthochitonidae (BP = 100, BPP = 1; Okusu et al. 2003; Sigwart et al. 2013), suggesting a revision of its placement within Mopaliidae. Consequently, our results recommend assigning *Nuttallochiton* to Acanthochitonidae and raising the genus *Plaxiphora* to the level of an independent family, Plaxiphoridae fam. nov., thereby resolving critical taxonomic inconsistencies within Acanthochitonina.

The diversification of *Acanthochitona* began during the Late Cretaceous period (92.05 Ma) and coincided with the marked shifts in sea levels that had occurred during this time. The Late Cretaceous is noted for elevated global temperatures and rising sea levels, extending the shallow marine habitats. These environmental shifts most likely contributed to the emergence of new ecological niches and the diversification of marine organisms, including chitons, boosting biodiversity. This era also saw the development of intricate marine ecosystems, facilitating an expansion in the diversity of marine invertebrates, particularly molluscs, which played a crucial role in shaping marine biodiversity (Smith et al. 2001).

Our molecular clock analysis dated the origin of the crown group of Polyplacophora to the Devonian (377.95 Ma). Such an estimate predates those from previous molecular and fossil-based studies, which had placed the origin during the Carboniferous period (Vinther et al. 2012, 2017). Notably, the earliest representatives of Neoloricata, characterized by an articulamentum extending into sutural laminae or sclerites, have been discovered in Carboniferous strata (Puchalski et al. 2008; Sirenko 2006). Our findings suggest that the initial divergence within Chitonina occurred during the Jurassic (191.84 Ma), with most families and subfamilies represented in our timetree diverging during the Cretaceous.

While the findings of our molecular clock analysis largely agree with those of previous work (Irisarri et al. 2020), the timing of Lepidochitonidae divergence within Acanthochitonina exhibited a distinction. Prior studies have shown Lepidochitonidae to have split from Mopaliidae (e.g., *K*. *tunicata*, *D*. *gothicus*, and *C*. *stelleri*) ~ 150 Ma, whereas our study indicates it to be much later, at ~ 220.48 Ma. Such a variation in estimates may be due to limitations in taxon sampling, potential inaccuracies in molecular clock models, or uncertainties in the calibration points used.

## Data Availability

The NCBI GenBank accession numbers for the mitochondrial genomes of the 5 *Acanthochitona* species are as follows: *A*. *achates* (PQ301026), *A*. *circellata* (PQ301027), *A*. *defilippii* (PP419021), *A*. *rubrolineata* (PQ301028), and *A*. *feroxa* sp. nov. (OR757114). The NCBI GenBank accession numbers for the *COI* sequences of *A*. *feroxa* sp. nov. are PQ651609–PQ651679. All data supporting the findings of this study are available within the article and its Supplementary Information.
